# Nutritional Assessment in Patients with Chronic Diseases: Tools, Challenges, and Future Directions

**DOI:** 10.3390/nu15224794

**Published:** 2023-11-16

**Authors:** Huiyu Tang, Ming Yang

**Affiliations:** 1 Center of Gerontology and Geriatrics, West China Hospital, Sichuan University, Chengdu 610041, China; 2 National Clinical Research Center for Geriatrics, West China Hospital, Sichuan University, Chengdu 610041, China

Chronic diseases, such as cardiovascular diseases, diabetes, cancer, and chronic respiratory diseases, are leading causes of morbidity and mortality worldwide [1]. According to the Global Burden of Disease, poor nutrition can exacerbate these conditions, leading to increased symptom severity, a reduced quality of life, and higher healthcare costs [2]. Conversely, adequate nutrition can help manage symptoms, improve treatment outcomes, and enhance the overall wellbeing of patients with chronic diseases.

Currently, the Global Leadership Initiative on Malnutrition (GLIM) guidelines [3] emphasize the critical importance of assessing nutrition, and addressing malnutrition, in patients with chronic diseases. Malnutrition, as defined by GLIM, includes two components: “(1) phenotypic criteria (e.g., unintentional weight loss, low body mass index [BMI], reduced muscle mass) and (2) etiologic criteria (e.g., reduced food intake or assimilation, inflammation, or disease burden)”. These criteria provide a standardized framework for assessing nutrition and identifying malnutrition in patients with chronic diseases, ensuring that healthcare providers consider both the physical manifestations and the underlying causes of malnutrition.

This editorial aims to provide a concise summary of the tools available for nutritional assessment in patients with chronic diseases, illuminate the unique challenges faced by clinicians and researchers in this area, and offer insights into the promising future directions in the field of nutritional assessment.

## 1. Nutritional Assessment Tools

Various tools and methods are available for nutritional assessment, each with its strengths and limitations. These tools include dietary surveys, anthropometric measurements, nutritional assessment questionnaires, biochemical markers, and medical devices such as dual-energy X-ray absorptiometry (DXA) and bioelectrical impedance analysis (BIA).

### 1.1. Dietary Surveys

Dietary surveys serve as important tools for assessing nutritional status. These surveys involve collecting comprehensive information about an individual’s dietary habits, food choices, portion sizes, and meal patterns. Through careful analysis, dietary surveys can provide valuable insights into macronutrient and micronutrient intake, as well as dietary patterns that may contribute to, or mitigate, chronic disease risk. Commonly used surveys include 24-h dietary recalls, food frequency questionnaires, and food diaries [4].

### 1.2. Anthropometric Measurements

Anthropometric measurements involve assessing physical characteristics, such as body weight, height, BMI, waist circumference, and skinfold thickness. These measurements are simple but very useful, as unintentional weight loss and reduced BMI remain the phenotypic criteria of GLIM-defined malnutrition [3]. 

### 1.3. Nutritional Assessment Questionnaires

Many questionnaires are available for assessing nutritional status. For example, the Subjective Global Assessment (SGA) was originally used in surgical patients to diagnose protein–calorie malnutrition but is now widely utilized in other conditions, such as in patients with HIV [5], cancer [6], and chronic renal disease [7]. 

Additionally, the Mini Nutritional Assessment (MNA) was developed to provide a single, rapid assessment of malnutrition risk in geriatric populations in hospitals, outpatient clinics, and nursing homes [8].

Other questionnaires, such as the Malnutrition Screening Tool (MST) [9], Malnutrition Universal Screening Tool (MUST) [10], Nutrition Risk Score-2002 (NRS-2002) [11], and Short Nutritional Assessment Questionnaire (SNAQ) [12], can also be utilized to evaluate nutritional status in different clinical settings.

### 1.4. Biomarkers

Biomarkers may offer objective data on a patient’s nutritional status. Common biomarkers include serum albumin, prealbumin, hemoglobin, and various vitamins and minerals [13]. These markers can indicate nutritional deficiencies or imbalances that may require intervention. Additionally, inflammation biomarkers, such as C-reactive protein, might be useful for identifying the etiologic criteria of malnutrition as recommended by the GLIM [3].

### 1.5. DXA and BIA

Incorporating DXA and BIA into the toolkit for nutritional assessment enhances the precision and depth of the evaluation. These techniques provide valuable insights into body composition, allowing healthcare providers to tailor interventions more effectively to address the specific nutritional needs and challenges of each patient.

In this Special Issue, two papers focus on the implementation of BIA among patients with chronic diseases. In a prospective study, Skórka and colleagues [14] reported that BIA was useful for the assessment and monitoring of nutritional status in patients with a coexisting chronic wound. Additionally, Bozic et al. [15] demonstrated that BIA was a non-invasive, simple tool for the fast and affordable detection of sarcopenia in patients with liver cirrhosis. 

## 2. Challenges in Nutritional Assessment

### 2.1. Disease-Specific Considerations

One of the foremost challenges in nutritional assessment lies in addressing disease-specific considerations. Each chronic disease presents unique nutritional requirements and challenges [1]. For example, heart failure patients must meticulously manage their sodium intake to prevent fluid retention and symptom exacerbation. In contrast, individuals with neurodegenerative disorders, such as Parkinson’s disease or Alzheimer’s disease, may struggle with dysphagia and oral health issues, requiring specialized approaches to ensure an adequate nutrient intake [16]. These disease-specific nuances demand careful consideration and individualized nutritional plans.

### 2.2. Comorbidity Burden

Geriatric patients often present with multiple chronic conditions, a phenomenon known as multimorbidity [17]. This comorbidity burden further complicates the assessment and management of nutrition. Healthcare providers must navigate the intricate interplay between these conditions, considering how one condition may influence the nutritional needs and challenges associated with another. For instance, a patient with both diabetes and heart disease may face conflicting dietary recommendations, necessitating a tailored approach that strikes a balance between glycemic control and sodium restriction.

### 2.3. Patient Variability

Nutritional needs and preferences can vary significantly among patients, even among those with the same chronic disease. Factors such as age, gender, genetics, cultural background, socioeconomic status, and individual dietary habits all contribute to this variability. For instance, dietary preferences may be influenced by cultural traditions or religious beliefs, leading to variations in food choices and meal preparation methods. This diversity underscores the need for healthcare providers to adopt a patient-centered approach, taking into account the individual’s unique circumstances and preferences when designing nutritional interventions.

### 2.4. Resource Limitations

Resource limitations pose a significant challenge to comprehensive nutritional assessment and intervention, particularly in healthcare settings with constraints on budget and personnel [18]. Time restrictions, inadequate access to specialized nutritional services, and limited reimbursement for nutritional care services can hinder healthcare providers’ ability to conduct thorough nutritional assessments and deliver tailored interventions. Addressing these resource limitations is crucial for ensuring that patients with chronic diseases receive the nutritional support that they need.

## 3. Future Directions in Nutritional Assessment

### 3.1. Personalized Nutrition

Advances in genomics and metabolomics hold significant promise for the development of personalized nutritional plans [19]. By analyzing an individual’s genetic makeup and metabolic profile, healthcare providers can adapt dietary recommendations to address specific nutrient needs and vulnerabilities. For example, genetic testing may reveal a heightened risk of vitamin D deficiency, prompting the inclusion of vitamin D-rich foods or supplements in the patient’s diet. Personalized nutritional approaches have the potential to revolutionize the field by providing highly targeted interventions that optimize health outcomes [20].

### 3.2. Digital Health Solutions

The advent of digital health technologies, including mobile applications, wearable devices, and telehealth platforms, presents new opportunities for real-time nutritional assessment and support. These tools enable individuals to monitor their dietary intake, track changes in nutritional status, and receive personalized feedback and recommendations from healthcare providers. For example, a smartphone app that records food intake and provides instant nutritional analysis can empower patients to make informed dietary choices and adhere to prescribed nutritional plans [21]. Moreover, telehealth consultations with registered dietitians can facilitate ongoing nutritional support, even in remote or under-served areas.

### 3.3. Interdisciplinary Collaboration

The complexity of managing chronic diseases necessitates a multidisciplinary approach toward care. Collaboration between healthcare professionals, including dietitians, nurses, physicians, pharmacists, physical therapists, and occupational therapists, can facilitate comprehensive and holistic nutritional assessments and care. Interdisciplinary teams can address the diverse and evolving needs of patients with chronic diseases effectively. 

### 3.4. Education and Training

To address the unique challenges of nutritional assessment in the context of chronic diseases, healthcare professionals must receive specialized education and training. This education should encompass not only the basics of nutritional assessment but also disease-specific considerations, communication strategies, cultural competence, and the integration of emerging technologies. An informed and skilled workforce is essential for delivering high-quality nutritional care to patients with chronic diseases, promoting evidence-based practice, and advancing the field of clinical nutrition.

In conclusion, nutritional assessment in patients with chronic diseases is a complex and multifaceted endeavor that requires a nuanced and patient-centered approach. The increasing prevalence of chronic diseases underscores the importance of addressing the unique challenges associated with nutritional assessment and management. Researchers, clinicians, policymakers, and educators should collaborate to develop and implement innovative tools and strategies that optimize nutritional care, improve health outcomes, and enhance the quality of life for individuals living with chronic diseases. 
